# Comparative Evaluation of the Effect of Different Oxygen Layer Inhibitors on the Thickness of the Oxygen-Inhibited Layer on Nanohybrid Resin Composite and Its Surface Microhardness: An In Vitro Study

**DOI:** 10.7759/cureus.114766

**Published:** 2026-08-18

**Authors:** Thanvi Shaji, Kavya Sabu, Sonu Ravindran, Sabir Muliyar, Suraj U, Krishnapriya N

**Affiliations:** 1 Conservative Dentistry and Endodontics, Malabar Dental College and Research Centre, Malappuram, IND

**Keywords:** composite resin, deox, mylar strip, nanohybrid composite, oxygen-inhibited layer, oxygen layer inhibitors, post-polymerization polishing, surface microhardness

## Abstract

Background

This study aimed to evaluate and compare the effect of oxygen layer inhibitors such as DeOx (Ultradent Products, Inc.), Mylar strip (Unident Denmed), and post-polymerization polishing (Shofu Super-Snap Rainbow Technique Kit) on the thickness of the oxygen-inhibited layer (OIL) on nanohybrid resin composite (Ivoclar Vivadent Tetric N-Ceram Syringe Refill Universal Composite Resin) and its microhardness.

Methodology

A total of 40 nanohybrid resin composite samples were shaped as discs of 6 mm diameter and 4 mm thickness. Oxygen layer inhibitors were divided into Group 1 (DeOx, n = 10), Group 2 (Mylar strip, n = 10), Group 3 (post-polymerization polishing, n = 10), and Group 4 (Control, n = 10). The samples were polymerized for 20 seconds with a light-emitting diode curing unit. The samples were then measured for OIL thickness at 50× magnification using a three-dimensional optical profilometer. Furthermore, each group of samples was evaluated for surface microhardness using an electronic Vickers hardness tester applying a 100 gf load for 10 seconds on the surface at different equidistant points and maintaining a minimum distance of 1 mm adjacent to the margins of the sample. The diagonal length of each notch was measured directly using a graduated ocular lens at 40× magnification. Data were analyzed using one-way analysis of variance, and Tukey’s post hoc test was applied for multiple pairwise comparisons. A p-value of less than 0.05 was considered statistically significant.

Results

The highest OIL film thickness was seen in the Control group (283.35 μm), followed by post-polymerization polishing (79.02 μm), Mylar strip (41.60 μm), and DeOx (23.09 μm). The DeOx group (86.48 VHN) demonstrated the highest mean microhardness values, followed by Mylar strip (78.36 VHN), post-polymerization polishing (62.88 VHN), and Control (51.64 VHN). These differences in values were statistically significant.

Conclusions

OIL thickness and surface microhardness were significantly affected by the application of DeOx, Mylar strip, and post-polymerization polishing. In this study, the application of DeOx resulted in reduced thickness of OIL and increased surface microhardness of the nanohybrid resin composite, followed by Mylar strip and post-polymerization polishing.

## Introduction

Composite restorations are widely used in dentistry due to their excellent esthetic and functional properties. Several mechanical characteristics of dental composites are crucial to the material’s durability, including compressive strength, tensile strength, bond strength, and hardness [1-4].

Light-activated polymerization is the most common method for curing dimethacrylate-based resin composites. This process involves the generation of free radicals that initiate and sustain the polymerization of resin monomers. However, when the composite is polymerized in the presence of atmospheric oxygen, the surface layer remains incompletely cured due to the formation of an oxygen-inhibited layer (OIL). Oxygen scavenges the free radicals required for polymerization, thereby limiting the degree of conversion at the surface. As a result, the OIL may negatively influence the mechanical performance of the composite, particularly by decreasing its surface microhardness.

Studies have shown that oxygen can either hinder or enhance vinyl polymerization [5]. When a new layer of the composite is added, the OIL formed on the previous layer easily accommodates the overlying material. These layers have significant implications on the properties of the composite. In some cases, it provides a beneficial feature, allowing chemical bonding between successive layers in multi-step restorative procedures [6-8]. However, unintentional or uncontrolled OIL formation can lead to reduced surface hardness, increased wear, and higher susceptibility to staining and plaque accumulation [7,9,10].

The thickness of the OIL has been reported to vary among different dimethacrylate-based resin composites. These differences are influenced by several material-related factors, including the type and concentration of filler particles as well as the cross-linked network density of the resin matrix. The incorporation of low-viscosity diluent monomers, such as triethylene glycol dimethacrylate, has also been shown to affect the extent of oxygen inhibition by altering monomer mobility and oxygen diffusion. Furthermore, in fiber-reinforced resin composites, both the presence of reinforcing fibers and their orientation within the composite can influence the depth of the OIL [11].

Nanotechnology is one of the recent advancements in composites, including the introduction of nanohybrid composites. Nanohybrid composites have a particle size of 0.4-5 µ and contain nanosized particles as well as conventional-sized filler particles. They can be used in both anterior and posterior restorations due to their better esthetic properties, good wear resistance, low polymerization shrinkage, and better compressive and tensile strength [1,12-14].

Various techniques have been employed to minimize the formation of the OIL by limiting the exposure of the resin surface to atmospheric oxygen during polymerization. One such approach involves the use of DeOx, a glycerin-based gel supplied in a syringe delivery system, which facilitates easy application, including in posterior composite restorations. Another commonly used method is the placement of a Mylar strip over the composite surface during light curing, which acts as a physical barrier to oxygen and results in a smoother, more completely polymerized surface. In addition, post-polymerization polishing has been shown to remove a substantial portion of the oxygen-inhibited surface layer, thereby enhancing the color stability and overall surface characteristics of composite restorations [9,15-17].

Optical profilometers are widely used to assess the surface morphology of various materials, such as optical components and biomaterials. The astigmatic approach, interferometry, focus detection, and other optical techniques have been used with optical profilometers. Unlike traditional contact profilometers that rely on a stylus physically touching the sample, optical profilometers use light-based techniques such as white light interferometry, structured illumination, or cone-focused microscopy to obtain detailed three-dimensional (3D) surface data without any physical contact [18].

Therefore, this study aimed to comparatively evaluate the effect of different oxygen layer inhibition techniques on the thickness of the OIL on nanohybrid resin composite and its surface microhardness, measured using a 3D optical profilometer and an electronic Vickers hardness tester.

## Materials and methods

Study design

This in vitro study was conducted in the Department of Conservative Dentistry and Endodontics at Malabar Dental College and Research Centre, Malappuram, Kerala, India. As the research was performed exclusively using commercially available restorative materials and did not use any human subjects, extracted teeth, patient data, animal tissues, or other biological specimens, approval from the Institutional Ethics Committee was not required.

Sample size estimation

The sample size was calculated based on the diametral tensile strength data reported by Hadi et al. [1]. An expected mean difference of 1.41 MPa and a pooled standard deviation of 0.975 MPa were considered for the calculation. Assuming a two-sided significance level of 5% (α = 0.05), 90% statistical power, and an equal allocation ratio, the estimated minimum sample size was 10 samples per group. As the study included four experimental groups, the final sample size was 40 samples (10 samples in each group). The sample size estimation was performed using the formula for comparison of two independent means, consistent with the methodology adopted by Hadi et al. [1].

Study materials

A total of 40 nanohybrid resin composite samples were prepared and allocated equally into the following four experimental groups (n = 10 each) for assessing OIL thickness and surface microhardness: Group 1, DeOx gel (Ultradent Products, Inc.); Group 2, Mylar strip (Unident Denmed); Group 3, polishing with Shofu Super-Snap Rainbow Technique Kit; and Group 4, Control. Each group comprised 10 disc-shaped nanohybrid resin composites of 6 mm diameter and 4 mm thickness. Samples were fabricated and polymerized according to the manufacturer’s guidelines to ensure consistency and limit experimental variability (Table 1).

**Table 1:** List of materials.

Materials	Brand
Nanohybrid composite resin	Ivoclar Vivadent Tetric N-Ceram Syringe Refill Universal Composite Resin
DeOx	Ultradent Products Inc.
Mylar strip	Unident Denmed
Polishing kit	Shofu Super-Snap Rainbow Technique Kit
LED light-curing unit	Ivoclar Bluephase N MC

Sample preparation

A total of 40 nanohybrid resin composite samples were fabricated under standardized laboratory conditions using a stainless steel mould of 6 mm diameter and 4 mm thickness. The composite resin was packed into the moulds as horizontal increments. Each sample was fabricated by curing successive 2 mm increments of composite resin using a Bluephase N MC LED curing unit (Ivoclar) with a light intensity of approximately 1,200 mW/cm². Every increment was exposed to the curing light for 20 seconds. Following the placement of the final increment, the samples were allocated into four groups (n = 10).

**Figure 1:**
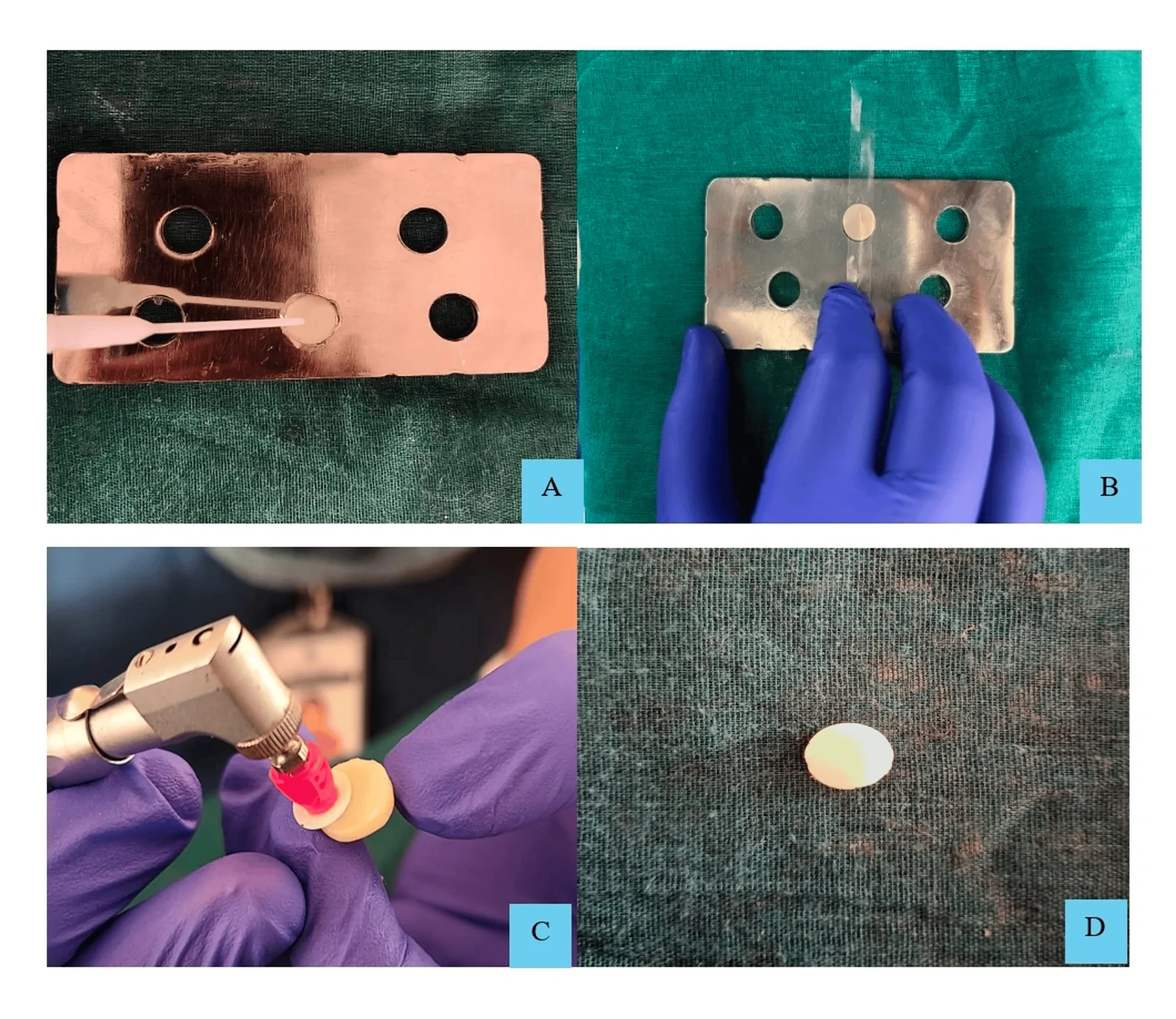
(A) DeOx. (B) Mylar strip. (C) Post-polymerization polishing. (D) Control.

For Group 1, DeOx gel was applied as a uniform layer over the surface of each of the 10 composite disc samples before light curing. In Group 2, a Mylar strip was carefully adapted over the surface of each sample before polymerization. Group 3 samples were light-cured and subsequently finished and polished using the Shofu Super-Snap Rainbow Technique Kit. Finishing was initially performed with the black (coarse) and violet (medium) discs, followed by polishing and super-polishing using the green (fine) and red (super-fine) discs, respectively. Group 4 (control) samples were light-cured without the use of any oxygen layer inhibitors (Figure 1).

After polymerization, all samples were examined under standardized lighting conditions by a single calibrated investigator to identify any surface imperfections, including voids, air bubbles, cracks, or other irregularities. The dimensions of the samples were measured using a digital Vernier caliper to ensure consistency. Only samples with uniform dimensions, complete polymerization, and no visible defects were selected for inclusion in the study. Following fabrication, all samples were stored individually in distilled water at 37°C for 24 hours before evaluation of the OIL thickness and surface microhardness.

Evaluation of oxygen-inhibited layer thickness and surface microhardness

**Figure 2:**
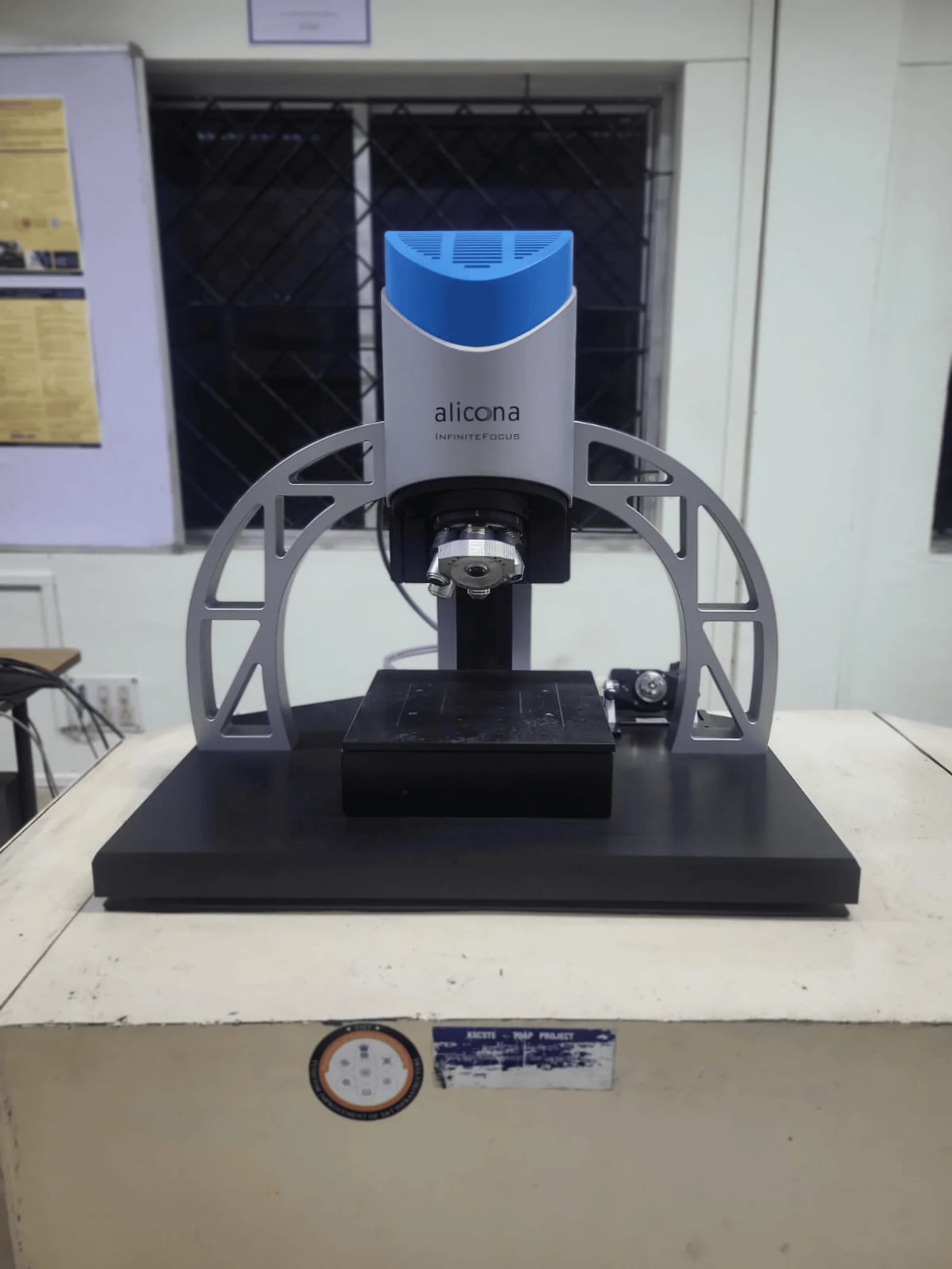
Three-dimensional optical profilometer.

**Figure 3:**
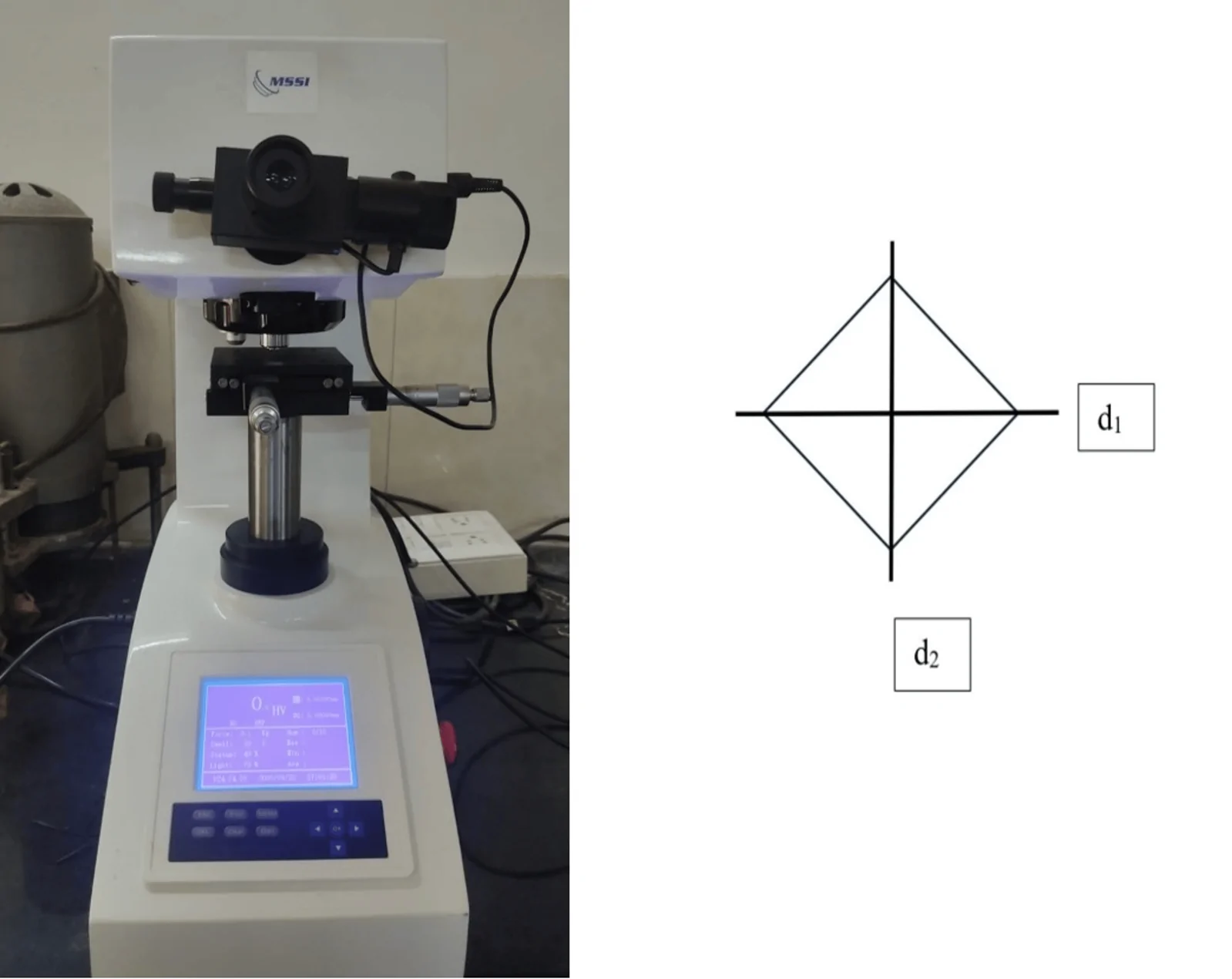
Electronic Vickers hardness tester.

The samples were measured for OIL thickness at 50× magnification using a 3D optical profilometer (Alicona Infinite Focus 5G) based on optical interferometry, which projects light onto the sample, and differences in reflected light create a highly precise 3D surface profile mapping the exact thickness across the entire surface (Figure 2). Furthermore, each group of samples was evaluated for surface microhardness using an electronic Vickers hardness tester (MSSI), applying a 100 g force load for 10 seconds on the surface at different equidistant points and maintaining a minimum distance of 1 mm adjacent to the margins of the sample. The diagonal length of each notch was measured directly using a graduated ocular lens at 40× magnification. Surface microhardness was measured using the following formula: HV = 1.8544 × F/d^2^, where HV is Vickers hardness, d is the average of the two diagonals (d1 and d2) of the imprint, and F is the applied load (Figure 3).

Statistical analysis

The collected data were entered and analyzed using SPSS version 26.0 (IBM Corp., Armonk, NY, USA). Descriptive statistics, including mean and standard deviation, were calculated for all groups. The normality of data distribution was assessed before inferential analysis. Intergroup comparisons were performed using one-way analysis of variance (ANOVA) to evaluate differences among the groups. Upon obtaining statistically significant results, Tukey’s post hoc test was applied for multiple pairwise comparisons. A p-value of less than 0.05 was considered statistically significant.

## Results

The present study demonstrated that oxygen layer inhibition methods significantly influence both OIL thickness and microhardness. The DeOx group exhibited the lowest OIL thickness and the highest microhardness, indicating superior performance. The Control group demonstrated the highest OIL thickness (283.35 ± 94.35), followed by post-polymerization polishing (79.02 ± 8.55), Mylar strip (41.60 ± 3.38), and DeOx (23.09 ± 3.76). One-way ANOVA indicated a statistically highly significant difference among the groups (F = 52.64, p < 0.001), confirming that the groups differed significantly in terms of OIL thickness (Table 2).

**Table 2:** Comparison of oxygen-inhibited layer thickness among groups.

Group	Mean	SD	F-value	P-value
DeOx	23.09	3.76	52.64	<0.001*
Post-polymerization polishing	79.02	8.55		
Mylar strip	41.60	3.38		
Control	283.35	94.35		

Tukey’s honestly significant difference (HSD) post hoc analysis for OIL thickness revealed statistically significant differences between all group pairs (p < 0.05), confirming substantial variation among treatments. The Control group exhibited significantly higher OIL thickness compared to DeOx (mean difference = 260.26, p < 0.001), post-polymerization polishing (mean difference = 204.33, p < 0.001), and Mylar strip (mean difference = 241.76, p < 0.001). The post-polymerization polishing group also demonstrated significantly greater OIL thickness compared to DeOx (mean difference = 55.93, p < 0.001) and Mylar strip (mean difference = 37.43, p < 0.001). Additionally, Mylar strip showed significantly higher OIL thickness than DeOx (mean difference = 18.51, p = 0.002). Overall, DeOx consistently exhibited the lowest OIL thickness, while Control showed the highest, with all differences being statistically significant (Table 3).

**Table 3:** Post hoc test (Tukey’s honestly significant difference) for oxygen-inhibited layer thickness.

Comparison	Mean difference	P-value	Significance
DeOx vs. control	-260.26	<0.001*	Significant
DeOx vs. post-polymerization polishing	-55.93	<0.001*	Significant
DeOx vs. Mylar strip	-18.51	0.002*	Significant
Mylar strip vs. control	-241.76	<0.001*	Significant
Mylar strip vs. post-polymerization polishing	-37.43	<0.001*	Significant
Post-polymerization polishing vs. control	-204.33	<0.001*	Significant

The mean microhardness values were highest in the DeOx group (86.48 ± 1.42), followed by Mylar strip (78.36 ± 1.50), post-polymerization polishing (62.88 ± 4.97), and Control (51.64 ± 3.84). One-way ANOVA revealed a statistically highly significant difference among the groups (F = 111.07, p < 0.001), indicating that the type of surface treatment had a significant effect on microhardness (Table 4).

**Table 4:** Comparison of microhardness among groups.

Group	Mean	SD	F-value	P-value
DeOx	86.48	1.42	111.07	<0.001*
Post-polymerization polishing	62.88	4.97		
Mylar strip	78.36	1.50		
Control	51.64	3.84		

The Tukey’s HSD post hoc analysis demonstrated statistically significant differences between all group comparisons (p < 0.05), indicating clear intergroup variation in microhardness. The DeOx group showed significantly higher microhardness compared to Control (mean difference = 34.84, p < 0.001), Mylar strip (mean difference = 8.12, p = 0.006), and post-polymerization polishing (mean difference = 23.60, p < 0.001). Furthermore, the Mylar strip group exhibited significantly greater hardness than Control (mean difference = 26.72, p < 0.001) and post-polymerization polishing (mean difference = 15.48, p < 0.001). The post-polymerization polishing group also showed significantly higher microhardness compared to Control (mean difference = 11.24, p = 0.0003). These findings indicate a consistent hierarchy in microhardness values across all groups, with DeOx demonstrating superior performance, followed by Mylar strip, post-polymerization polishing, and Control (Table 5).

**Table 5:** Post hoc test (Tukey’s honestly significant difference) for microhardness.

Comparison	Mean difference	P-value	Significance
DeOx vs. Control	34.84	<0.001*	Significant
DeOx vs. Mylar strip	8.12	0.006*	Significant
DeOx vs. post-polymerization polishing	23.60	<0.001*	Significant
Mylar strip vs. control	26.72	<0.001*	Significant
Mylar strip vs. post-polymerization polishing	15.48	<0.001*	Significant
Post-polymerization polishing vs. control	11.24	0.0003*	Significant

**Figure 4:**
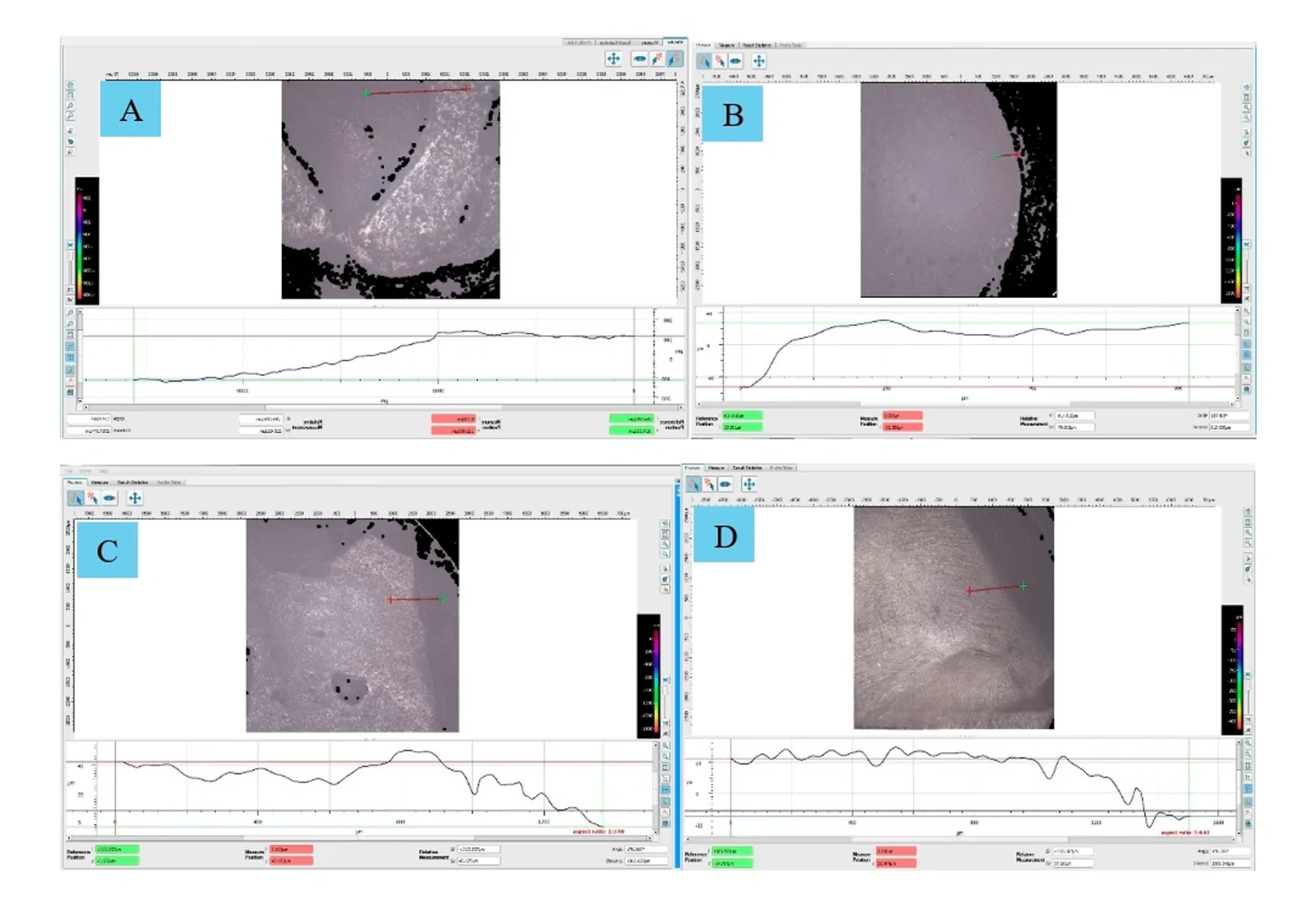
Three-dimensional profilometric images. (A) DeOx group. (B) Mylar strip group. (C) Post-polymerization polishing group. (D) Control group.

Three-dimensional optical profilometric images are shown in Figure 4.

**Figure 5:**
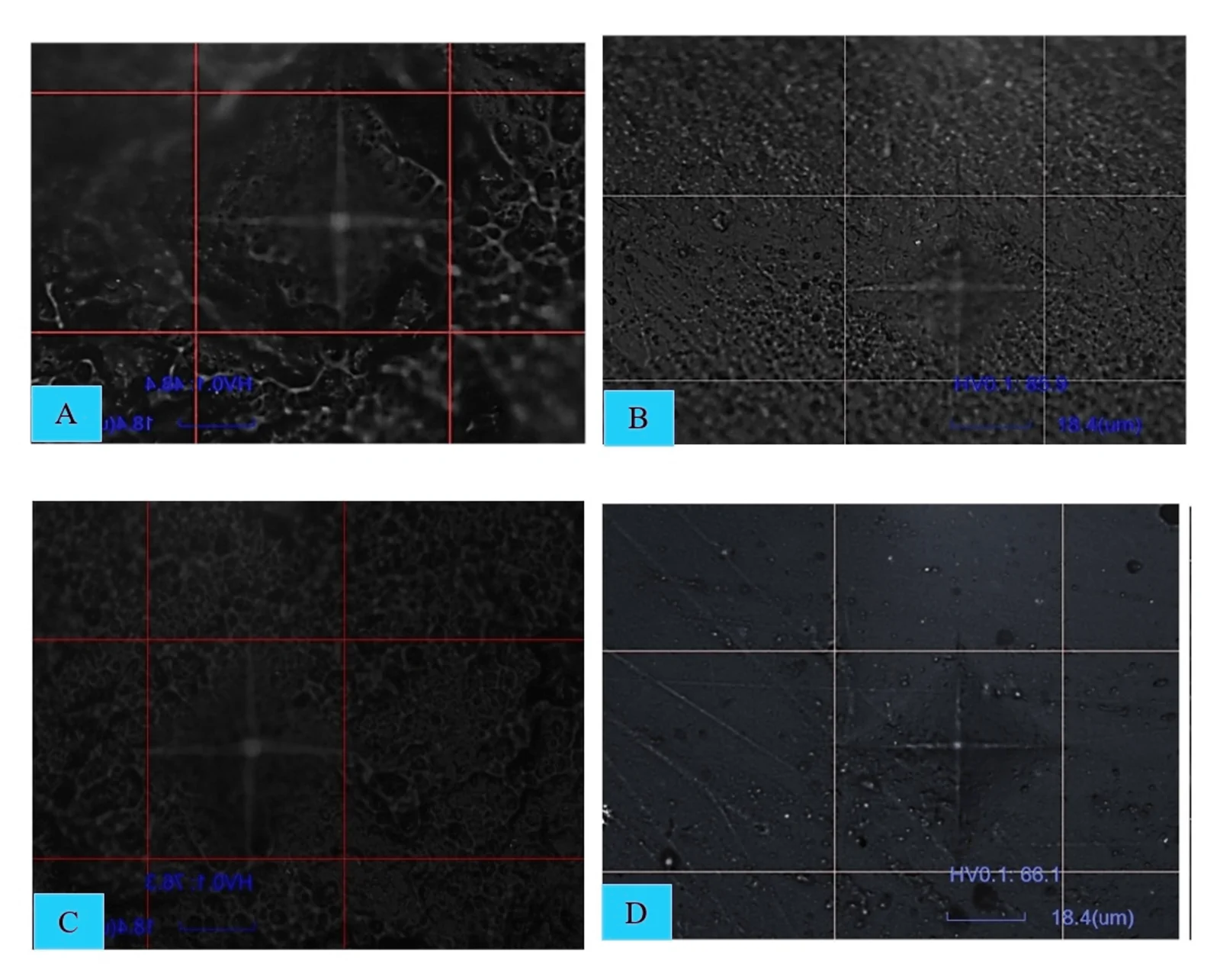
Microhardness measurement images. (A) DeOx group. (B) Mylar strip group. (C) Post-polymerization polishing group. (D) Control group.

 Microhardness measurement images are shown in Figure 5.

## Discussion

Incremental placement of resin composites is widely recommended to minimize polymerization shrinkage and improve the degree of monomer conversion. In such multilayer restorative procedures, the strength of the bond between successive composite increments is critical to the long-term performance of the restoration. Several studies have reported that the OIL contributes positively to interlayer adhesion by providing unreacted monomers that facilitate chemical bonding between adjacent increments [19]. Kim et al. (2006) further demonstrated that when the oxygen inhibition layer is absent or excessively thin, the limited availability of unreacted monomers may compromise interfacial chemical bonding, thereby reducing the ability of the restoration to withstand polymerization shrinkage stresses [20].

The thickness of the composite increment has been identified as one of the most critical determinants of polymerization efficiency. According to Rueggeberg et al. (1988), there is a marked decline in the degree of conversion when the increment thickness exceeds 2.5 mm. Consequently, the incremental layering technique was advocated in this study to prepare the samples [21].

Gugnani et al. in their study stated that the Bluephase N curing light unit can cure composites up to a depth of 2 mm when cured for 20 seconds, reaching the adequate depth of cure according to the International Society of Automation standards [22]. In this study, DeOx, the glycerine-based gel, demonstrated superior performance in reducing OIL thickness with 23.09 μm, followed by the Mylar strip group (41.60 μm), and the polishing group (79.02 μm). Increased OIL thickness was noted in the Control group, with a thickness of 283.35 μm.

The result obtained from this study could be because DeOx, when used before polymerization, acts as a physical barrier that adapts perfectly to the surface of the composite, thus eliminating oxygen, which reacts with free radicals. However, the Mylar strip merely acts as a physical barrier and cannot adapt well, as DeOx gel causes more entrapment of atmospheric oxygen, thereby increasing the formation of OIL compared to DeOx. Improper placement of the Mylar strip also contributed to more oxygen inhibition layer formation. This result is in accordance with the findings of Hadi et al. [1]. Post-polymerization finishing and polishing only partially remove the oxygen inhibition layer, which is inferior to DeOx and the Mylar strip [7].

Surface microhardness is one of the core properties of resin composites to enhance clinical durability. Surface microhardness improves wear and tear resistance, avoids microfractures, reduces plaque retention, and decreases the formation of secondary caries. The reduction of the oxygen inhibition layer by DeOx enhanced surface polymerization by increasing the cross-linking density, leading to a better degree of conversion and resulting in increased surface microhardness of the composite resin [23,24]. DeOx showed increased surface microhardness at 86.48 VHN compared to the Mylar strip (78.36 VHN) and polishing (62.88 VHN) groups. The lowest surface microhardness was noted in the control group at 51.64 VHN.

In the nanohybrid composite, due to better surface characteristics and optimized filler particle distribution, oxygen diffusion is reduced, thus decreasing the formation of the oxygen inhibition layer [12,13]. The verdict is that the use of DeOx in nanohybrid composite restoration reduces the thickness of the oxygen inhibition layer and increases surface microhardness, which leads to increased clinical durability and longevity of nanohybrid composite restoration.

The limitations of this in vitro study include its inability to fully simulate the oral environment, the evaluation of only one nanohybrid resin composite and a single LED curing protocol, and the exclusion of other commercially available oxygen inhibition layer inhibitors.

## Conclusions

In this study, DeOx significantly reduced the thickness of the oxygen inhibition layer and thereby increased surface microhardness, followed by the Mylar strip and polishing. This contributes to increased longevity and clinical durability of nanohybrid composite restorations, making it a valuable step in achieving optimal restorative outcomes.
